# Effects of pH and Cultivation Time on the Formation of Styrene and Volatile Compounds by *Penicillium expansum*

**DOI:** 10.3390/molecules24071333

**Published:** 2019-04-04

**Authors:** Hye Won Kim, Sang Mi Lee, Jeong-Ah Seo, Young-Suk Kim

**Affiliations:** 1 Department of Food Science and Engineering, Ewha Womans University, Seoul 120-750, Korea; tttyio1234@naver.com (H.W.K.); smlee78@ewha.ac.kr (S.M.L.); 2 School of Systems Biomedical Science, Soongsil University 369 Sangdo-ro, Dongjak-gu, Seoul 06978, Korea; sja815@ssu.ac.kr

**Keywords:** styrene, phenylalanine metabolism, *Penicillium expansum*, volatile compounds, pH, cultivation time

## Abstract

Styrene can be formed by the microbial metabolism of bacteria and fungi. In our previous study, styrene was determined as a spoilage marker of Fuji apples decayed by *Penicillium expansum*, which is responsible for postharvest diseases. In the present study, *P. expansum* was cultivated in potato dextrose broth added with phenylalanine—which is a precursor of styrene—using different initial pH values and cultivation times. Volatile compounds were extracted and analyzed using gas chromatography-mass spectrometry (GC-MS) combined with stir-bar sorptive extraction. The 76 detected volatile compounds included 3-methylbutan-1-ol, 3-methyl butanal, oct-1-en-3-ol, geosmin, nonanal, hexanal, and γ-decalactone. In particular, the formation of 10 volatile compounds derived from phenylalanine (including styrene and 2-phenylethanol) showed different patterns according to pH and the cultivation time. Partial least square-discriminant analysis (PLS-DA) plots indicated that the volatile compounds were affected more by pH than by the cultivation time. These results indicated that an acidic pH enhances the formation of styrene and that pH could be a critical factor in the production of styrene by *P. expansum*. This is the first study to analyze volatile compounds produced by *P. expansum* according to pH and cultivation time and to determine their effects on the formation of styrene.

## 1. Introduction

Styrene has been found in diverse foods such as beef meat, cereals, coffee beans, fruits, apple-based alcoholic beverages, and wheat beer [1,2,3]. The presence of styrene in foods can adversely affect their aroma due to its strong pungent and unpleasant odor [4]. Styrene can originate from food packaging materials [5] as well as the natural metabolism of raw agricultural materials [1]. It can also be formed by the microbial metabolism of bacteria [6] and fungi [7]. Several studies have investigated the production of styrene by fungi such as *Pichia carsonii* [8], *Fusarium oxysporum* [7], *Penicillium citrinum* [9], and *Penicillium expansum* [10]. Among them, *P. expansum* is a filamentous fungus that is widely found in certain types of spoiled fruits such as apples and plums [11] and is well known to produce styrene [12]. This fungus is responsible for the blue mold that is a major postharvest disease of apples [11,13]. This disease, related to styrene formation, can also result in off-flavors of processed apple products [14].

Several studies have demonstrated that volatile compounds are produced by *P. expansum*. Karlshøj et al. profiled the volatile compounds produced by apples decayed by *P. expansum* using an electronic-nose device [12]. Those authors detected 50 volatile compounds, among which volatile compounds such as styrene, 3-methyl-1-butanol, 3-methyl-1-butyl acetate, and 1-methoxy-3-methylbenzene were determined as being fungal biomarkers of *P. expansum*. Additionally, Kim et al. (2018) showed that the levels of ethanol, 3-methylbutan-1-ol, benzaldehyde, acetaldehyde, styrene, ethyl acetate, ethyl 2-methylbutanoic acid, and ethyl octanoate were significantly elevated in Fuji apples decayed by *P. expansum* [15].

The formation of volatile compounds by a fungus can be affected by various cultivation conditions, including the culture medium composition, temperature, and pH [16]. In particular, Miao et al. determined that the pH of the cultivation medium could significantly affect the formation of secondary volatile compounds [16]. Furthermore, Lee et al. determined that the production of volatile compounds by *Saccharomycopsis fibuligera* KJJ81 depends on the cultivation time [17]. However, there has been only one report on the effects of various culture media on the formation of volatile compounds by *P. expansum* [18]. That study found that cultivating *P. expansum* on various media such as pine leaves, pine stems, pine wood, mature dark bark, and potato dextrose broth (PDB) resulted in the production of different volatile compounds, including styrene.

While some previous studies have investigated the formation of styrene by *P. expansum*, the critical effects of culturing conditions have not been elucidated. Therefore, the objectives of this study were to (a) investigate the critical factors affecting the formation of styrene by cultivating *P. expansum* on PDB using different pH values and cultivation times and (b) profile the overall volatile compounds produced by *P. expansum* in cultivation media.

## 2. Results and Discussion

### 2.1. Analysis of Volatile Compounds of P. expansum According to pH and Cultivation Time

The volatile compounds produced by *P. expansum* are listed in Table 1. The 76 volatile compounds identified comprised 4 acids, 15 alcohols, 11 aldehydes, 11 benzenes, 9 esters, 7 furans, 6 hydrocarbons, 9 ketones, 3 nitrogen-containing compounds, and 1 sulfur-containing compound. Volatile fungal compounds can be produced via primary and secondary metabolism involving numerous precursors such as amino acids, fatty acids, and carbohydrates [19]. The present study detected diverse alcohols such as butan-1-ol, 3-methylbutan-1-ol, 2-ethylhexna-1-ol, and octan-1-ol. In particular, the well-known fungal volatile compound 3-methylbutan-1-ol [12,20] was detected at a higher level than other alcohols throughout the cultivation period. Other 3-methyl branched-chain volatiles such as 3-methyl butanal were also detected. Both 3-methylbutan-1-ol and 3-methyl butanal are commonly generated from leucine [19,20]. Reduction by alcohol dehydrogenase can convert 3-methyl butanal into 3-methylbutan-1-ol [21]. The levels of these volatile compounds derived from leucine were higher at pH 9 than at pH 5, which suggests that the leucine metabolism of *P. expansum* was more strongly activated at an alkaline pH.

C8 aliphatic compounds such as octanol, octenol, and octanone are characteristic fungal volatile compounds [25,26,27,28,29]. The present study found diverse C8 compounds such as oct-1-en-3-ol, octan-1-ol, octan-3-one, and octanal. Oct-1-en-3-ol, which is also called mushroom-flavor alcohol, was detected in all of the samples, and its level peaked after 24 hours of cultivation.

A musty off-flavor and odor note is given by (4S,4aS,8aR)-4,8a-dimethyl-1,2,3,4,5,6,7,8-octahydronaphthalen-4a-ol (geosmin) to fish [30], dry beans [31], and red table beets [32], and it can be synthesized by fungi [33], bacteria [34], and algae [35]. When Mattheis and Roberts (1992) cultivated P. expansum in Czapek agar, they identified geosmin as a major volatile compound [36]. Geosmin is known to be derived from isoprenoid. It has been demonstrated that isopentenyl diphosphate, which is a major intermediate in the synthesis of isoprenoid, can be produced via the mevalonate pathway or the methylerythritol phosphate pathway [37] and possibly also the pentose phosphate pathway [38]. The present study detected geosmin only when culturing at pH 5, indicating that the formation of geosmin by P. expansum was significantly affected by the cultivation pH.

Aldehydes can be produced from various precursors such as amino acids, carbohydrates, and fatty acids [39]. Some aldehydes such as decanal, nonanal, and hexanal were detected at higher levels than the other aldehydes. Korpi et al. found nonanal to be one of the main microbial volatile aldehydes in laboratory culture experiments, although it was not reported in field samples [40]. Hexanal, which is a straight long-chain aldehyde, can be formed from long-chain fatty acids such as palmitic acid and stearic acid via enzymatic oxidation [41]. In addition, hexan-1-ol can be converted reversibly into hexanal by alcohol dehydrogenase [42]. The level of hexanal was higher than that of hexan-1-ol in all of the present cultivation samples.

Most ketones are generated by lipid oxidation via β-oxidation of free fatty acids during microbial metabolism. Some ketones such as octan-3-one, 6-methylhept-5-en-2-one, and 5-hexyloxolan-2-one (γ-decalactone) were detected in this study, with octan-3-one only being identified at pH 5. This ketone has a musty and mushroom odor note and is reportedly a microbial volatile organic compound [43] that can be formed via the aerobic oxidation of linolenic acid and linoleic acid [41]. The precursors of γ-decalactone included oleic acid, linoleic acid, and other unsaturated fatty acids. In the first of three steps, ricinoleic acid is formed through the hydroxylation of oleic acid. Then, 4-hydroxy decanoic acid is formed via the reduction of ricinoleic acid from acetyl CoA (acetyl coenzyme A). The last step is lactonization, in which 4-hydroxy decanoic acid is converted into γ-decalactone [44,45].

A particularly interesting finding of this study was that the level of styrene was significantly elevated throughout the cultivation period at pH 5, whereas this tendency was not observed at pH 9. Other volatile compounds derived from phenylalanine also showed characteristic patterns of formation according to pH and cultivation time. Therefore, this study compared the contents of volatile compounds derived from phenylalanine in *P. expansum* according to pH and cultivation time.

### 2.2. Effects of pH and Cultivation Time on the Formation of Styrene and Volatile Compounds Derived from Phenylalanine

**Figure 1:**
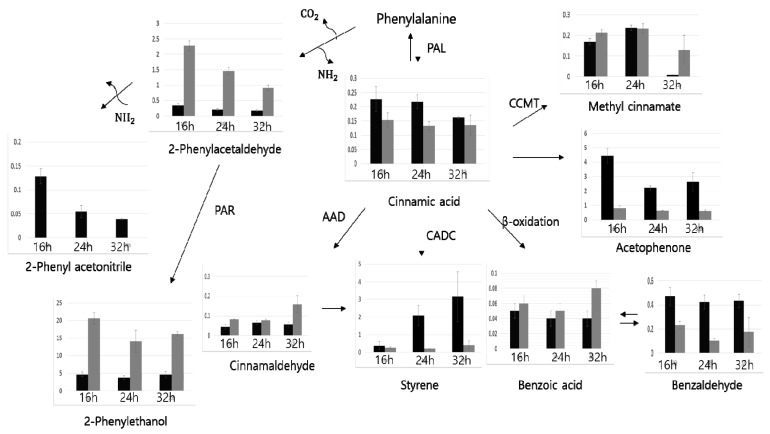
The contents of volatile compounds derived from phenylalanine at different pH and cultivation times and their possible pathways in *P. expansum*. Notes: All values are mean values of relative peak area to that of internal standard ± standard deviation from three replicates. PAL—phenylalanine ammonia lyase, CADC—cinnamic acid decarboxylase, PAR—phenyl acetaldehyde reductase, CCMT—cinnamic acid carboxyl methyl transferase, AAD—aryl-aldehyde dehydrogenase. Error bars represent standard deviation of three replicates.

*P. expansum* was cultivated at different pH values and cultivation times. Figure 1 shows the volatile compounds derived from phenylalanine at different pH values and cultivation times and the possible pathways involved in their generation.

Phenylalanine can be converted into cinnamic acid as the primary product of phenylalanine degradation by phenylalanine ammonia-lyase (PAL). Fungi can then participate in the conversion of cinnamic acid into styrene by cinnamic acid decarboxylation [46]. The amount of styrene produced in the present study was significantly higher after 24 hours of cultivation at pH 5 than after 16 hours. Additionally, the level of cinnamic acid peaked after 16 hours of cultivation at pH 5 and thereafter tended to decrease. It seems that cinnamic acid—which is a highly efficient precursor of styrene—is rapidly converted into styrene as soon as cinnamic acid is synthesized. In particular, *Penicillium* strains are well known to have the ability to form styrene from cinnamic acid [8,47]. This means that PAL, which can convert phenylalanine into cinnamic acid, might be a critical enzyme for the formation of styrene. In addition, Pagot et al. reported that the synthesis of styrene by PAL was strongly activated during the exponential phase in *Penicillium* strains [48]. The peaking of cinnamic acid after 16 hours (in the exponential phase) at pH 5 could therefore be related to the synthesis of a considerable amount of PAL. Accordingly, the formation of styrene by *P. expansum* was elevated at pH 5. On the other hand, both styrene and cinnamic acid were detected at much lower levels at pH 9 than at pH 5, and their levels did not increase significantly with the cultivation time. This also could be related to the activity of PAL, which is a reversible enzyme. The activity of PAL can be markedly affected by pH. The ability of PAL to convert cinnamic acid into phenylalanine (reverse reaction) is high at an alkaline pH, and, accordingly, the production of phenylalanine peaks [49]. As a result, an alkaline pH could result in the decreased production of styrene.

Moreover, 2-phenylethanol, which has fruity, floral, and rose-like odor notes [50,51,52,53], was another major volatile compound derived from phenylalanine. First, 2-phenylacetaldehyde is produced via the decarboxylation and deamination of phenylalanine, and then 2-phenylethanol is biosynthesized from 2-phenylacetaldehyde by phenyl acetaldehyde reductase [47]. The amount of 2-phenylethanol formed was considerably greater at pH 9 than at pH 5 in the present study. Many bacteria and fungi respond to a high extracellular pH by synthesizing deaminase that hydrolyzes amino acids [53,54]. Furthermore, Ghosh et al. identified that an alkaline pH enhances the production of aromatic alcohols [54]. Those authors found that the formation of three aromatic alcohols (tryptophol, 2-phenylethanol, and tyrosol) by *Candida albicans* was threefold higher under an alkaline condition. Accordingly, the production of a large amount of 2-phenylethanol at pH 9 in *P. expansum* in the present study could have been induced by the alkaline pH. In addition, other volatile compounds derived from phenylalanine such as phenyl acetaldehyde, 2-phenylacetonitrile, benzaldehyde, acetophenone, benzoic acid, and cinnamaldehyde were also detected. Among them, benzaldehyde was detected at a higher level at pH 5 than at pH 9, and its level peaked after 16 hours of cultivation. On the other hand, 2-phenylacetonitrile derived from phenylacetaldehyde was only detected at pH 5, which might have been due to all phenylacetaldehydes being converted into 2-phenylethanol at pH 9. In summary, volatile compounds derived from phenylalanine produced by *P. expansum* could be considerably affected by the extracellular pH and cultivation time.

**Figure 2:**
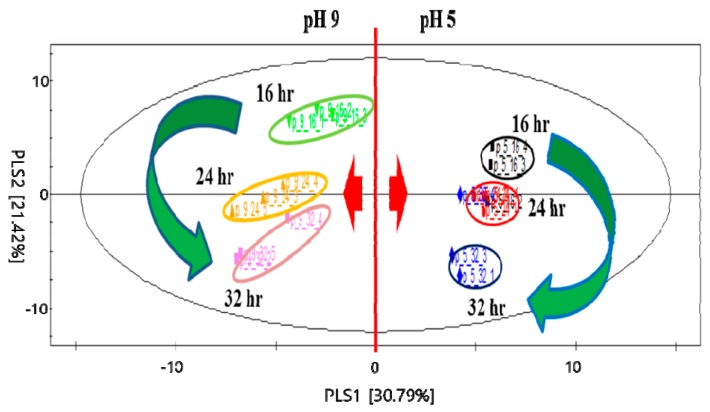
Partial least square-discriminant analysis (PLS-DA) score plot of volatile compounds produced by *P. expansum* according to pH and cultivation time.

Partial least square-discriminant analysis (PLS-DA) was conducted to determine the differences in volatile compounds produced by *P. expansum* and the significant effects of pH and cultivation time on the formation of volatile compounds. Figure 2 shows the PLS-DA score plot for the comparison of volatile compounds produced by *P. expansum*. PLS (Partial least square) component 1 (PLS 1) and PLS component 2 (PLS 2) explained 30.79% and 21.42% of the variance, respectively, and hence together explained 52.21% of the total variance. The parameters of the cross-validation modeling were component 5, R2X = 0.73, R2Y = 0.65, and Q2Y = 0.40. A permutation test involving 100 iterations was also conducted to validate the model, which yielded R2 = 0.25 and Q2 = −0.58.

All of the samples at pH 5 and 9 were located on the positive and negative PLS 1 axes, respectively, while all of the samples cultivated for 16 and 32 hours were located on the positive and negative PLS 2 axes, respectively. As the cultivation time increased, the samples moved along PLS 2. Table 2 and Table 3 list the major volatile compounds (with a criterion of the variable importance plot (VIP) > 0.8) identified in *P. expansum*.

The negative PLS 1 axis was related to most of the aldehydes and alcohols, while the positive PLS 1 axis was related to some benzenes such as styrene, benzaldehyde, and 1,3,5-trimethylbenzene, while styrene was also associated with the negative PLS 2 axis. These results demonstrated that the formation of styrene could be considerably influenced by an acidic pH and a longer cultivation time in *P. expansum*. In addition, 2-phenylethanol was positioned on the negative PLS 1 axis, which indicates that it could be affected by an alkaline pH in *P. expansum*. In addition, Figure 2 shows that the formation of volatile compounds by *P. expansum*, including styrene, could be affected more by the pH than by the cultivation time.

## 3. Materials and Methods

### 3.1. Chemicals

Potato dextrose broth (PDB) was obtained from Becton Dickinson (Dickinson and Company, Sparks, MD, USA). Phenylalanine was purchased from Samchun Chemicals (Pyeongtaek-si, Gyeonggi-do, Korea). The 2,3,5-trimethyl pyrazine was purchased from Sigma-Aldrich (St. Louis, MO, USA). Methanol was obtained from J.T.Baker (Phillipsburg, NJ, USA). Authentic standard compounds for positive identification of volatile compounds were purchased as follows: *N*,*N*-dibutylformamide, 2-phenylpropan-1-ol, pentadecan-2-one, propan-2-yl hexadecanoate, 3,4-dimethylphenyl methanol, hexadecan-1-ol, and 3-phenylfuran-2,5-dione were purchased from Alfa Aesar (Haverhill, MA, USA); methyl 2-(3-oxo-2-pentylcyclopentyl)acetate, 3-methyl-2H-furan-5-one, and 6-methylheptan-2-one were obtained from SejinCI (Seoul, Korea), while all of the other authentic standards were purchased from Sigma-Aldrich (St. Louis, MO, USA).

### 3.2. Strain and Cultivation of Penicillium expansum

*P. expansum* HR5-4 was isolated from naturally decayed apples. *P. expansum* was identified as previously reported [55] and cultivated in 40 mL of PDB media contained 0.1% phenylalanine. One milliliter of spore suspension (10^7^ spores/mL) of *P. expansum* was inoculated in a 250 mL Erlenmeyer flask with screw cap and placed in a shaking incubator (Vision Scientific Co., Ltd., Bucheon-si, Gyeonggi-do, Korea) at 25 °C and 180 rpm. *P. expansum* was cultivated at different cultivation times (16, 24, and 32 hours) and initial pH (pH 5 and pH 9). Each cultivation time was determined by growth phase of *P. expansum* (16 hours: Exponential phase, 24 and 32 hours: Stationary phase). Initial pH of media was adjusted by using 0.1 M HCl and NaOH.

### 3.3. Analysis of Volatile Compounds Using Gas Chromatography-Mass Spectrometry (GC-MS)

#### 3.3.1. Extraction of Volatile Compounds

After vacuum filtration, aliquots (8 mL) of cultivation media were transferred into a 10 mL glass vial (Agilent Technologies, Santa Clara, CA, USA). Volatile compounds were extracted using a polydimethylsiloxane-coated stir bar (PDMS twister 10 mm length, 1.0 mm film thickness) (GERSTEL GmbH and Co. KG, Mülheim an der Ruhr, Germany). The PDMS twister was placed in samples and stirred at 1000 rpm and ambient temperature for 60 min. After extraction, PDMS twister was washed with distilled water and dehydrated with lint-free tissue paper. Then, PDMS twister was placed in desorption liner tube (GERSTEL GmbH and Co.) and inserted into a thermal desorption unit (TDU). Volatile compounds were desorbed by increasing the temperature of the TDU. The initial temperature of 40 °C was maintained for 0.5 min. After that, the temperature increased at a rate of 120 °C/min to 220 °C and held for 5 min. With cooled injection system (CIS), cryo-focusing temperature was maintained at −60 °C using liquid N_2_ supply and the temperature of the CIS-4 PTV (Programmed Temperature Vaporizer) was increased thermally to 250 °C at a rate of 10 °C/sec and held for 3 min. The temperatures of transfer line and ion source were 280 and 250 °C, respectively. The splitless mode was used as injection mode.

#### 3.3.2. GC-MS Analysis

Volatile compounds were analyzed using an Agilent 7890B gas chromatograph (GC) connected to a 5977A mass detector (Agilent Technologies, Santa Clara, CA, USA). A Stabilwax column (30 m length × 0.25 mm internal diameter × 0.25 μm film thickness, Restek Corporation, Bellefonte, PA, USA) was used. The oven temperature was initiated at 40 °C (5 min), increased to 220 °C at a rate of 4 °C/min and then held at 220 °C (10 min). Helium was used as carrier gas at a constant flow rate of 0.8 mL/min. The mass spectrum was obtained in EI (electron ionization) mode at 70 eV, mass scan rate of 4.5 scans/sec, and mass scan range of 35–350 *m/z*. In addition, the analysis of some volatile compounds, which could be derived from phenylalanine, were conducted using selective ion monitoring (SIM) mode. The list of volatile compounds measured and the SIM qualifying ions are presented in Table 4.

#### 3.3.3. Identification and Semiquantification of Volatile Compounds

Volatile compounds were positively identified by comparing their mass spectral data and retention index (RI) values with those of authentic standard compounds. Otherwise, the other volatile compounds, whose authentic standard compounds were not available, were tentatively identified by comparing with the mass spectral libraries (Wiley 9th edition and NIST, version 08) and retention index (RI) values in published literatures [52,53,54]. The retention index (RI) values were calculated using n-alkanes from C_7_ to C_30_ as external standards. The semiquantification of volatile compounds was calculated by their peak areas to that of internal standard compound. Five microliters of 2,3,5-trimethyl pyrazine (100 mg/L in methanol) were used as an internal standard.

### 3.4. Statistical Analysis

Partial least square-discriminant analysis (PLS-DA) was performed to determine the differences of volatile compounds of *P. expansum* according to pH and cultivation time and the significant effect on the formation of volatile compounds using SIMCA-P (version 11.0, Umetrics, Umea, Sweden). Analysis of variance (ANOVA) was also conducted with general linear model procedure in SPSS (version 12.0, Chicago, IL, USA) to evaluate significant differences of volatile compounds in samples. In order to evaluate significant differences (*p* < 0.05), Duncan’s multiple range test was conducted.

## 4. Conclusions

This study investigated volatile compounds produced by *P. expansum* according to pH and cultivation time. A total of 76 volatile compounds such as 3-methylbutan-1-ol, 3-methyl butanal, oct-1-en-3-ol, geosmin, nonanal, hexanal, and γ -decalactone were detected. In particular, the formation of volatile compounds derived from phenylalanine such as styrene showed characteristic patterns according to pH and cultivation time. In particular, the level of styrene was considerably higher at pH 5 than at pH 9. Moreover, as cultivation time increased, the production of styrene significantly increased at pH 5. On the other hand, styrene was detected at much lower level at pH 9 than at pH 5, and also its level was not significantly increased according to cultivation time. On PLS-DA plots, the formation of volatile compounds of *P. expansum* was more highly affected by pH condition than by cultivation time. As a result, the cultivation pH could be a critical factor in the production of styrene in *P. expansum*. This study is a first report on the analysis of volatile compounds according to pH and cultivation time and determines their effects on the formation of styrene in P. expansum.

## Figures and Tables

**Table 1:** The contents of volatile compounds of *Penicillium expansum* at different pH and cultivation times.

No. ^1)^	RI ^2)^	Volatile Compounds	pH	Relative Peak Area ^3)^			ID ^5)^
				Cultivation Times ^4)^			
				16 h	24 h	32 h	
**Acids**							
a1	1465	Acetic acid	5	0.06 ± 0.01ab ^6)^	0.07 ± 0.01b	0.04 ± 0.01a	A
			9	0.07 ± 0.05 a	0.03 ± 0.01a	0.04 ± 0.02a	
a2	>2200	Benzoic acid	5	0.07 ± 0.02a	0.04 ± 0.02a	0.04 ± 0.01a	A
			9	0.09 ± 0.01b	0.06 ± 0.01a	0.07 ± 0.01ab	
a3	>2200	Hexadecanoic acid	5	0.24 ± 0.04a	0.30 ± 0.13a	0.26 ± 0.02a	A
			9	0.27 ± 0.09ab	0.25 ± 0.01a	0.40 ± 0.02b	
a4	>2200	(E)-3-Phenylprop-2-enoic acid (Cinnamic acid)	5	0.24 ± 0.06b	0.10 ± 0.03a	0.05 ± 0.01a	A
			9	0.25 ± 0.04a	0.23 ± 0.06a	0.37 ± 0.05a	
**Alcohols**							
ac1	1152	Butan-1-ol	5	0.13 ± 0.01b	0.10 ± 0.03ab	0.09 ± 0.01a	A
			9	0.08 ± 0.02a	0.10 ± 0.04a	0.06 ± 0.01a	
ac2	1210	3-Methylbutan-1-ol	5	0.90 ± 0.09a	0.96 ± 0.07a	0.95 ± 0.07a	A
			9	1.38 ± 0.14a	1.36 ± 0.07a	1.42 ± 0.19a	
ac3	1356	Hexan-1-ol	5	0.01 ± 0.00a	0.02 ± 0.00a	0.02 ± 0.01a	A
			9	0.02 ± 0.00a	0.01 ± 0.00a	0.02 ± 0.00b	
ac4	1456	Oct-1-en-3-ol	5	0.2 ± 0.02a	0.21 ± 0.05a	0.20 ± 0.01a	A
			9	0.06 ± 0.04a	0.20 ± 0.04b	0.07 ± 0.02a	
ac5	1493	2-Ethylhexan-1-ol	5	0.80 ± 0.12a	0.78 ± 0.17a	0.79 ± 0.14a	A
			9	0.96 ± 0.09a	1.16 ± 0.23a	1.06 ± 0.10a	
ac6	1562	Octan-1-ol	5	0.08 ± 0.01a	0.14 ± 0.02b	0.09 ± 0.02a	A
			9	0.09 ± 0.03a	0.22 ± 0.07b	0.10 ± 0.02a	
ac7	1625	2-(2-Ethoxyethoxy)Ethanol	5	0.08 ± 0.03a	0.09 ± 0.01a	0.09 ± 0.03a	A
			9	0.08 ± 0.01a	0.15 ± 0.01b	0.15 ± 0.02b	
ac8	1644	5-Methyl-2-propan-2-ylcyclohexan-1-ol	5	0.14 ± 0.03a	0.12 ± 0.01a	0.13 ± 0.01a	A
			9	0.15 ± 0.03a	0.15 ± 0.01a	0.17 ± 0.03a	
ac9	1830	(4S,4aS,8aR)-4,8a-Dimethyl-1,2,3,4,5,6,7,8-octahydronaphthalen-4a-ol (Geosmin)	5	0.02 ± 0.01a	0.22 ± 0.14b	0.33 ± 0.04b	B
			9	N.D. ^7)^ a	N.D. a	N.D. a	
ac10	1853	(2E)-3,7-Dimethylocta-2,6-dien-1-ol (Geraniol)	5	0.34 ± 0.04b	0.27 ± 0.03b	0.18 ± 0.02a	A
			9	0.12 ± 0.01a	0.12 ± 0.01a	0.10 ± 0.01a	
ac11	1920	2-Phenylethanol	5	2.68 ± 0.29a	3.19 ± 1.69a	2.70 ± 0.38a	A
			9	11.07 ± 0.89b	8.49 ± 0.75a	9.46 ± 0.77ab	
ac12	1937	2-Phenylpropan-1-ol	5	N.D. a	N.D. a	N.D. a	A
			9	0.20 ± 0.01b	0.12 ± 0.01a	0.12 ± 0.06a	
ac13	1971	Dodecan-1-ol	5	0.59 ± 0.08a	0.81 ± 0.08a	0.92 ± 0.20a	A
			9	0.72 ± 0.04a	0.98 ± 0.05ab	1.25 ± 0.20b	
ac14	>2200	3,4-Dimethylphenyl methanol	5	0.08 ± 0.02a	0.12 ± 0.01a	0.10 ± 0.02a	A
			9	N.D. a	N.D. a	N.D. a	
ac15	>2200	Hexadecan-1-ol	5	0.32 ± 0.10a	1.02 ± 0.23b	0.67 ± 0.20ab	A
			9	0.40 ± 0.07a	1.57 ± 0.28b	1.31 ± 0.39b	
**Aldehydes**							
ah1	<1000	3-Methylbutanal	5	N.D. a	N.D. a	N.D. a	A
			9	0.04 ± 0.00b	0.02 ± 0.00a	0.02 ± 0.00a	
ah2	<1000	Pentanal	5	0.05 ± 0.02a	0.07 ± 0.01a	0.09 ± 0.02a	A
			9	0.04 ± 0.01a	0.10 ± 0.02b	0.08 ± 0.02ab	
ah3	1091	Hexanal	5	0.13 ± 0.03a	0.21 ± 0.07a	0.83 ± 0.63a	A
			9	0.09 ± 0.05a	0.61 ± 0.42a	0.52 ± 0.40a	
ah4	1189	Heptanal	5	0.14 ± 0.03a	0.15 ± 0.02a	0.15 ± 0.04a	A
			9	0.12 ± 0.02a	0.23 ± 0.04b	0.20 ± 0.01b	
ah5	1292	Octanal	5	0.18 ± 0.03a	0.26 ± 0.03a	0.29 ± 0.07a	A
			9	0.15 ± 0.06a	0.64 ± 0.14b	0.26 ± 0.06a	
ah6	1397	Nonanal	5	0.73 ± 0.15a	2.37 ± 0.75b	1.16 ± 0.35a	A
			9	0.64 ± 0.25a	4.21 ± 1.58b	1.37 ± 0.20a	
ah7	1503	Decanal	5	0.63 ± 0.06a	1.04 ± 0.28a	0.80 ± 0.20a	A
			9	0.53 ± 0.33a	1.67 ± 0.43b	0.81 ± 0.02a	
ah8	1533	Benzaldehyde	5	3.40 ± 0.29b	1.67 ± 0.11a	1.95 ± 0.46a	A
			9	0.59 ± 0.09a	0.53 ± 0.06a	0.49 ± 0.07a	
ah9	1652	2-Phenylacetaldehyde	5	0.08 ± 0.02a	0.05 ± 0.03a	0.04 ± 0.01a	A
			9	0.81 ± 0.06c	0.57 ± 0.06b	0.25 ± 0.04a	
ah10	1820	(2E,4E)-Deca-2,4-dienal	5	0.17 ± 0.03a	0.21 ± 0.03a	0.40 ± 0.26a	A
			9	N.D. a	N.D. a	N.D. a	
ah11	2054	(E)-3-Phenylprop-2-enal (Cinnamaldehyde)	5	0.04 ± 0.01a	0.043 ± 0.02ab	0.08 ± 0.02b	A
			9	0.07 ± 0.00a	0.08 ± 0.01a	0.27 ± 0.18a	
**Benzenes**							
b1	<1000	Benzene	5	0.01 ± 0.01a	0.05 ± 0.04a	0.02 ± 0.00a	A
			9	0.03 ± 0.02a	0.06 ± 0.01a	0.08 ± 0.09a	
b2	1043	Toluene	5	0.07 ± 0.01a	0.07 ± 0.01a	0.07 ± 0.01a	A
			9	0.06 ± 0.01a	0.08 ± 0.01a	0.07 ± 0.01a	
b3	1130	Ethylbenzene	5	0.06 ± 0.03ab	0.13 ± 0.04b	N.D. a	A
			9	0.09 ± 0.02c	0.03 ± 0.01b	N.D. a	
b4	1143	1,4-Xylene	5	0.03 ± 0.00a	0.05 ± 0.03a	0.02 ± 0.00a	A
			9	N.D. a	N.D. a	N.D. a	
b5	1261	Styrene	5	0.34 ± 0.22a	2.09 ± 0.60ab	3.11 ± 1.40b	A
			9	0.24 ± 0.07a	0.23 ± 0.04a	0.39 ± 0.21a	
b6	1283	1,3,5-Trimethylbenzene	5	0.04 ± 0.03ab	0.07 ± 0.01b	0.01 ± 0.00a	A
			9	0.08 ± 0.01b	0.03 ± 0.03a	N.D.a	
b7	1748	Naphthalene	5	0.082 ± 0.02a	0.093 ± 0.017a	0.113 ± 0.019a	A
			9	0.066±0.005a	0.104 ± 0.005b	0.148 ± 0.018c	
b8	1938	2-Phenylacetonitrile	5	0.14 ± 0.04b	0.06 ± 0.02a	0.04 ± 0.00a	A
			9	N.D. a	N.D. a	N.D. a	
b9	1964	1,3-Benzothiazole	5	0.03 ± 0.00a	0.03 ± 0.00a	0.01±0.01a	A
			9	N.D. a	N.D. a	N.D. a	
b10	2017	Phenol	5	0.07 ± 0.04a	0.03 ± 0.01a	0.04 ± 0.00a	A
			9	0.18 ± 0.06a	0.04 ± 0.01a	0.10 ± 0.02a	
b11	>2200	Diphenylmethanone (Benzophenone)	5	0.07 ± 0.02b	0.04 ± 0.02ab	0.04 ± 0.01a	A
			9	0.15 ± 0.08a	0.08 ± 0.00a	0.11 ± 0.01a	
**Esters**							
e1	1488	6-Methylheptyl prop-2-enoate	5	0.02 ± 0.00a	0.02 ± 0.00a	0.02 ± 0.01a	A
			9	0.01 ± 0.00a	0.01 ± 0.00a	0.02 ± 0.00b	
e2	1755	2-Ethylhexyl 2-ethylhexanoate	5	0.18 ± 0.07a	0.294 ± 0.069a	0.28 ± 0.13a	B
			9	0.16 ± 0.04a	0.33 ± 0.01b	0.43 ± 0.04c	
e3	1873	(3-Hydroxy-2,2,4-trimethylpentyl) 2-Methylpropanoate	5	0.83 ± 0.12b	0.58 ± 0.13ab	0.45 ± 0.08a	C
			9	0.47 ± 0.03a	0.53 ± 0.03a	0.52 ± 0.17a	
e4	2087	Methyl (E)-3-phenylprop-2-enoate (Methyl cinnamate)	5	0.26±0.02ab	0.44 ± 0.14b	0.16 ± 0.03a	A
			9	0.32 ± 0.02a	0.36 ± 0.01a	0.27 ± 0.13a	
e5	>2200	Methyl hexadecanoate	5	N.D. a	N.D. a	N.D. a	A
			9	0.07±0.01a	0.09 ± 0.01ab	0.12 ± 0.03b	
e6	>2200	Propan-2-yl hexadecanoate	5	N.D. a	N.D. a	N.D. a	A
			9	0.05 ± 0.01a	0.29 ± 0.02c	0.11 ± 0.01b	
e7	>2200	Ethyl hexadecanoate	5	0.02 ± 0.01a	0.03 ± 0.01a	0.03 ± 0.00a	A
			9	0.03 ± 0.01a	0.04 ± 0.01b	0.03 ± 0.00a	
e8	>2200	Methyl 2-(3-oxo-2-pentylcyclopentyl)acetate	5	0.08 ± 0.03a	0.09 ± 0.01a	0.15 ± 0.05a	A
			9	0.08 ± 0.02a	0.12 ± 0.01ab	0.17 ± 0.06b	
e9	>2200	Dodecyl octanoate	5	0.04 ± 0.01a	0.13 ± 0.03b	0.10 ± 0.02b	C
			9	0.04 ± 0.01a	0.16 ± 0.02b	0.15 ± 0.07b	
**Furans**							
f1	1233	2-Pentylfuran	5	N.D. a	0.01 ± 0.00a	0.04 ± 0.02b	A
			9	0.01 ± 0.00a	0.02 ± 0.01ab	0.05 ± 0.02b	
f2	1475	Furan-2-carbaldehyde	5	0.11 ± 0.02b	0.05 ± 0.01a	0.076 ± 0.03ab	A
			9	0.06 ± 0.07a	0.04 ± 0.01a	0.04 ± 0.00a	
f3	1670	Furan-2-ylmethanol	5	0.12 ± 0.03a	0.12 ± 0.09a	0.76 ± 0.72a	A
			9	0.23 ± 0.04ab	0.19 ±0.06a	0.34 ± 0.05b	
f4	1730	3-Methyl-2H-furan-5-one	5	0.07 ± 0.04a	0.03 ± 0.00a	0.04 ± 0.00a	A
			9	0.06 ± 0.06a	0.04 ± 0.01a	0.06 ± 0.01a	
f5	1863	3-Phenylfuran	5	0.01 ± 0.01a	0.01 ± 0.02a	N.D.a	A
			9	12.76 ± 2.10a	11.92 ± 1.38a	15.18 ± 1.62a	
f6	>2200	3-Phenylfuran-2,5-dione	5	N.D. a	N.D. a	N.D. a	C
			9	0.12 ± 0.01a	0.06 ± 0.02a	0.07 ± 0.01a	
f7	>2200	5-(Hydroxymethyl) Furan-2-carbaldehyde (5-Hydroxymethyl-furfural)	5	0.33 ± 0.07b	0.09 ± 0.01a	0.09 ± 0.01a	A
			9	0.16 ± 0.05a	0.11 ± 0.01a	0.24 ± 0.15a	
**Hydrocarbons**							
h1	1000	Decane	5	0.04 ± 0.03ab	0.07 ± 0.01b	0.01 ± 0.01a	A
			9	0.08 ± 0.01b	0.02 ± 0.01a	0.01 ± 0.00a	
h2	1058	2-Methyldecane	5	N.D. a	N.D. a	N.D. a	B
			9	0.07± 0.02a	0.05 ± 0.01a	0.01 ± 0.01b	
h3	1200	Dodecane	5	N.D. a	N.D. a	N.D. a	A
			9	0.05 ± 0.01b	0.03 ± 0.00a	0.02 ± 0.00a	
h4	1600	Hexadecane	5	0.19 ± 0.03a	0.26 ± 0.02a	0.32 ± 0.10a	A
			9	0.24 ± 0.04a	0.25 ± 0.02a	0.40 ± 0.00b	
h5	1700	Heptadecane	5	0.18 ± 0.06a	0.26 ± 0.03a	0.25 ± 0.09a	A
			9	0.15 ± 0.02a	0.27 ± 0.02b	0.34 ± 0.03c	
h6	1798	Octadecane	5	0.31 ± 0.09a	0.27 ± 0.03a	0.27 ± 0.05a	A
			9	0.26 ± 0.05a	0.24 ± 0.02a	0.30 ± 0.03a	
**Ketones**							
k1	1265	Octan-3-one	5	0.02 ± 0.00a	0.09 ± 0.03b	0.02 ± 0.01a	A
			9	N.D. a	N.D. a	N.D. a	
k2	1240	6-Methylheptan-2-one	5	N.D. a	N.D. a	N.D. a	A
			9	0.01 ± 0.00a	0.02 ± 0.00b	0.01 ± 0.00ab	
k3	1287	Octan-2-one	5	N.D. a	N.D. a	N.D. a	A
			9	0.02 ± 0.01a	0.07 ± 0.00b	0.06 ± 0.02b	
k4	1307	1-Hydroxypropan-2-one	5	0.30 ± 0.00a	0.32 ± 0.08a	0.48 ± 0.08b	A
			9	0.32 ± 0.13a	0.35 ± 0.03a	0.42 ± 0.06a	
k5	1342	6-Methylhept-5-en-2-one	5	0.05 ± 0.02a	0.02 ± 0.00a	0.11 ± 0.03b	A
			9	0.01 ± 0.01a	0.03 ± 0.02a	0.15 ± 0.04b	
k6	1660	1-Phenylethanone (Acetophenone)	5	0.23 ± 0.07a	0.21 ± 0.02a	0.24 ± 0.05a	A
			9	0.21 ± 0.04a	0.21 ± 0.02a	0.24 ± 0.02a	
k7	1860	(5E)-6,10-Dimethylundeca-5,9-dien-2-one	5	0.1 ± 0.02a	0.14 ± 0.01a	0.25 ± 0.10a	A
			9	N.D. a	N.D. a	N.D. a	
k8	2023	Pentadecan-2-one	5	0.04 ± 0.01a	0.03 ± 0.01a	0.04 ± 0.00a	A
			9	N.D. a	N.D. a	N.D. a	
k9	2155	5-Hexyloxolan-2-one (γ–Decalactone)	5	0.16 ± 0.11a	0.07 ± 0.03a	0.18 ± 0.02a	A
			9	0.08± 0.00a	0.08 ± 0.00a	0.20± 0.06a	
**N-containing compounds**							
n1	1252	1,3-Thiazole	5	0.03 ± 0.01a	0.02 ± 0.01a	0.04 ± 0.02a	A
			9	0.02 ± 0.01a	0.02 ± 0.01a	0.03 ± 0.02a	
n2	1777	*N*,*N*-Dibutylformamide	5	0.28 ± 0.04b	0.35 ± 0.02b	0.20 ± 0.03a	A
			9	0.16 ± 0.04a	0.23 ± 0.02ab	0.29 ± 0.07b	
n3	1989	Quinoline	5	N.D. a	N.D. a	N.D. a	A
			9	0.09 ± 0.00b	0.08 ± 0.01b	0.07 ± 0.01a	
**S-containing compounds**							
s1	1080	(Methyldisulfanyl) Methane (Dimethyl disulfide)	5	0.06 ± 0.02a	0.02 ± 0.01a	0.07 ± 0.09a	A
			9	0.03 ± 0.00b	N.D. a	N.D. a	

Notes: ^1)^ All volatile compounds are listed by the order of their RI values in chemical class. ^2)^ Retention indices were determined using n-alkanes C_7_–C_30_ as external standards on a Stabilwax column. ^3)^ Mean values of relative peak area to that of internal standard ± standard deviation from three replicates. ^4)^ Cultivation times: 16 h: 16 hours, 24 h: 24 hours, 32 h: 32 hours. ^5)^ Identification of the compounds was based on the following: A, mass spectrum and retention index agreed with those of authentic compounds under the same conditions (positive identification); B, mass spectrum and retention index were consistent with those from NIST (National Institute of Standards and Technology) database (tentative identification) and literatures [22,23,24]; C, mass spectrum was consistent with that of W9N08 (Wiley and NIST) and manual interpretation (tentative identification). ^6)^ Significant differences (*p* < 0.05) between samples according to cultivation time using Duncan’s multiple comparison test. ^7)^ N.D. = not detected.

**Table 2:** The major volatile compounds identified in *P. expansum* according to variable importance plot (VIP > 0.8) list for PLS (Partial least square) component 1 of PLS-DA.

Volatile Compounds	VIP Value
**Positive direction**	
1,3,5-Trimethylbenzene	1.09
Ethylbenzene	1.08
Pentadecan-2-one	1.02
Decane	0.99
1,3-Benzothiazole	0.98
Benzaldehyde	0.98
Styrene	0.83
**Negative direction**	
3-Methylbutanal	1.50
Pentanal	1.42
2-Methyldecane	1.40
Heptanal	1.36
Dodecane	1.29
3-Methylbutan-1-ol	1.27
2-Pentylfuran	1.18
6-Methylheptan-2-one	1.17
Octan-2-one	1.14
Octanal	1.14
Hexan-1-ol	1.12
Nonanal	1.12
Decanal	1.10
Octan-1-ol	1.09
Hexadecane	1.09
2-(2-Ethoxyethoxy) Ethanol	1.08
2-Phenylacetaldehyde	1.08
Heptadecane	1.08
Naphthalene	1.07
2-Ethylhexyl 2-ethylhexanoate	1.05
3-Phenylfuran	1.02
2-Phenylethanol	1.01
2-Phenylpropan-1-ol	1.01
Dodecan-1-ol	1.00
Quinoline	0.99
(E)-3-Phenylprop-2-enal (Cinnamaldehyde)	0.98
Methyl (E)-3-phenylprop-2-enoate (Methyl cinnamate)	0.98
Hexadecan-1-ol	0.90
Dodecyl octanoate	0.90
Benzoic acid	0.88
Diphenylmethanone (Benzophenone)	0.84
Cinnamic acid	0.81

**Table 3:** The major volatile compounds identified in *P. expansum* according to variable importance plot (VIP > 0.8) list for PLS component 2 of PLS-DA.

Volatile Compounds	VIP Value
**Positive direction**	
2-Phenylpropan-1-ol	1.17
Dodecane	1.14
1,3,5-Trimethylbenzene	1.09
Ethylbenzene	1.08
Methyl (E)-3-phenylprop-2-enoate(Methyl cinnamate)	1.02
(2E)-3,7-Dimethylocta-2,6-dien-1-ol (Geraniol)	0.96
**Negative direction**	
Octan-1-ol	1.42
Decanal	1.40
6-Methylheptan-2-one	1.36
2-Pentylfuran	1.09
Pentanal	1.08
Octan-2-one	1.06
Hexadecane	1.05
Heptanal	1.04
Pentadecan-2-one	1.02
Octan-3-one	1.00
Heptadecane	0.99
Benzaldehyde	0.98
2-Ethylhexyl 2-ethylhexanoate	0.98
(E)-3-Phenylprop-2-enal (Cinnamaldehyde)	0.97
Hexan-1-ol	0.974
1,3-Thiazole	0.88
(2E,4E)-Deca-2,4-dienal	0.84
Styrene	0.83
6-Methylheptyl prop-2-enoate	0.81

**Table 4:** The list of phenylalanine degradation compounds measured and selected ions for qualification and quantification.

Volatile Compounds	Qualifier Ions (*m/z*)	Quantifier Ions (*m/z*)	ID ^1)^
Styrene	78, 103, 104	104	A
Benzaldehyde	77, 105, 106	106	A
Phenyl acetaldehyde	91, 92, 120	91	A
Acetophenone	77, 105, 120	105	A
2-Phenylethanol	91, 92, 122	91	A
2-Phenylacetonirtile	90, 116, 117	117	A
Cinnamaldehyde	103, 131	131	A
Methyl cinnamate	103, 131, 162	131	A
Benzoic acid	122, 105	105	A
Cinnamic acid	103, 147	147	A

^1)^ Identification of the compounds was based on the following: A, mass spectrum and retention index agreed with those of authentic compounds under the same conditions (positive identification); B, mass spectrum and retention index were consistent with those from NIST (National Institute of Standards and Technology) database (tentative identification) and literatures [22,23,24]; C, mass spectrum was consistent with that of W9N08 (Wiley and NIST) and manual interpretation (tentative identification).
